# T Helper Cell Subsets and Their Functions in Common Bottlenose Dolphins (*Tursiops truncatus*)

**DOI:** 10.3389/fimmu.2019.01578

**Published:** 2019-08-20

**Authors:** Sylvain De Guise, Milton Levin, Lindsay Jasperse, Guillermo Risatti, Randall S. Wells

**Affiliations:** ^1^Department of Pathobiology and Veterinary Science, University of Connecticut, Mansfield, CT, United States; ^2^Connecticut Sea Grant College Program, University of Connecticut, Groton, CT, United States; ^3^Sarasota Dolphin Research Program, Chicago Zoological Society and Mote Marine Laboratory, Sarasota, FL, United States

**Keywords:** T helper, Th1, Th2, Treg, cytokines, polarization, dolphin, immunology

## Abstract

Considerable efforts have been made to better understand the immune system of bottlenose dolphins in view of the common environmental challenges they encounter, such as exposure to polychlorinated biphenyls, oil spills, or harmful algal bloom biotoxins. However, little is known about the identity and functionality of the Th1, Th2, and Treg T helper cell subsets in bottlenose dolphins. The present study aimed at validating assays and reagents to identify T helper cell subsets and their functions in a subset of dolphins from Sarasota Bay, Florida, USA, which have been long studied and often used as a reference population. A population of CD4+ FOXP3+ lymphocytes was identified representing an average <1% of blood lymphocyte population, which is in the range observed in for Treg cells in other species. The use of porcine reagents to measure TGFß, one of the key Treg cytokines, was further validated using the relatively high-throughput and highly standardized Luminex technology. The proportion of circulating Treg cells was not correlated with the serum concentrations of the Treg effector cytokines TGFß and IL-10, nor could it significantly contribute to predicting the variability of T lymphocyte proliferation, suggesting that not all dolphin circulating Treg cells are functional and active. However, stimulation of dolphin lymphocytes with TGFß and IL-2 increased the expression of the gene for TGFß and IL-10, and stimulation with IL-12 and IFNγ induced a robust increase in the expression of the gene for IFNγ, suggesting the potential for polarization and differentiation of dolphin T helper cells toward a Treg and Th1 response, respectively. The lack of an increase in the expression of the genes for the Th2 cytokines IL-4 and IL-13 upon stimulation with IL-4 may be due to the requirement for IL-2 for a Th2 polarization as described in mice. However, regression analysis and PCA suggested the potential ability of both the Th1 and Th2 response to be triggered upon acute inflammatory signals. These results may be useful in better understanding the mechanisms by which the dolphin immune system is affected upon exposure to environmental challenges and how it responds to pathogen challenges.

## Introduction

Considerable efforts have been made to better understand the immune system of marine mammals in general and of common bottlenose dolphins (*Tursiops truncatus*) in particular (1), in view of the common environmental challenges they encounter, such as exposure to polychlorinated biphenyls or PCBs (2), oil spills (3), or harmful algal bloom biotoxins (4). However, little is known about the identity and functionality of different T helper cell subsets in these species.

It has long been recognized that naïve T cells can differentiate into T helper 1 (Th1) or T helper 2 (Th2) cells that play an important role in the adaptive immune system. The commitment toward a Th1 response is promoted by interferon gamma (INFγ) and interleukin (IL)-12, and Th1 cells in turn secrete INFγ, which stimulates cell-mediated immunity to help combat intracellular pathogens (e.g., viruses), eliminate cancerous cells, and stimulate delayed-type hypersensitivity (DTH) skin reactions, while at the same time inhibiting Th2 differentiation (5). The commitment toward a Th2 response is promoted by IL-4, and Th2 cells in turn produce IL-4, IL-5, IL-6, and IL-13, which inhibit cell mediated (Th1) immunity and promote humoral (i.e., antibody mediated) immune responses to help combat extracellular pathogens (e.g., extracellular bacteria, parasites) (5). However, important additional Th cell subsets have more recently been recognized.

T regulatory (Treg) cells are specialized CD4+ T cells that function to maintain self-tolerance and immune homeostasis by suppressing the activation, proliferation, and effector functions of various immune cells (6). In humans, alterations in the number and function of Treg cells have been implicated in many diseases such as type I diabetes (7), graft vs. host disease (8), systemic lupus erythematosus (9), and rheumatoid arthritis (10). Treg cells can be identified based upon cell surface expression of CD4^+^CD25^+^CD127^low^ or by the intracellular transcription factor Forkhead box P3 (FOXP3) (11). The commitment toward a Treg response is promoted by TGFß, and Treg cells in turn secrete IL-10 and TGFß, which down-regulate immune responses (11, 12).

Several functions of the immune system in general, and of T lymphocytes in particular, are modulated by the balance of cytokines secreted by different T cell subsets. The differentiation and regulation of naive T cells into different T cell subsets with specific functions are in turn modulated by a number of critical signals including cell to cell interactions and cytokines (12). A recent report suggested the potential for dysregulation of the Th1/Th2 balance and changes in T lymphocyte proliferation that might be attributable to effects on Treg in bottlenose dolphins following exposure to oil after the *Deepwater Horizon* oil spill, but more in-depth studies were hindered by the lack of validated methods and reagents in this species.

The present study aimed to identify and assess the functions of Th1, Th2, and Treg cells in bottlenose dolphins. It clearly identified Treg cells from dolphin blood and their serum cytokines, demonstrated the functionality of Th1 and Treg dolphin cells, and assessed relationships among serum cytokines in wild bottlenose dolphins.

## Materials and Methods

### Source of Blood

Twenty long-term resident bottlenose dolphins from Sarasota Bay, FL, United States, were captured in June 2018, sampled, and released as part of health assessment programs (which included the immunological data presented here), as previously described in detail elsewhere (13, 14). The dolphins sampled included 10 males and 10 females, ranging in age from 2 to 48 years old (mean 17 years old), and likely represented a good cross section of the population sampled. Whole blood was collected in Vacutainer tubes (Becton Dickinson, Franklin Lakes, New Jersey, USA) with sodium heparin as part of the routine physical examinations, kept cool on ice packs and shipped overnight for functional immunological assays. In addition, 1 ml serum from each of those 20 dolphins was collected and immediately frozen prior to shipping on dry ice for cytokine analysis. Dolphin samples were collected under National Marine Fisheries Service Scientific Research Permit No. 20455, issued to RSW, as approved by the Mote Marine Laboratory Institutional Animal Care and Use Committee (IACUC). All samples were received and experiments performed following approval from the University of Connecticut IACUC. Human whole blood was purchased from Biological Specialties Corporation (Colmar, PA 18915, USA) and bovine and ovine whole blood were purchased from Lampire Biological Laboratory (Pipersville, PA 18947, USA). Blood purchased from commercial sources was deemed exempt from IACUC oversight by the University of Connecticut IACUC.

### Isolation of Peripheral Blood Mononuclear Cells

Dolphin blood samples were processed immediately upon receipt in the laboratory, within 24 h of collection. Blood samples from other species were also processed immediately upon receipt in the laboratory, however the collection time of samples from commercial sources was not known. Dolphin, human, bovine, and ovine whole blood was diluted 1:1 with phosphate buffered saline (PBS) with 2 mM EDTA (Miltenyi, Auburn, CA 95602, USA), layered on top of an equal volume of Ficoll-Paque Plus 1.077 (GE, Pittsburgh, PA 15264, USA), and centrifuged for 40 min at 400 g, as per manufacturer's instructions. The peripheral blood mononuclear cell (PBMC) layer was collected, washed twice with Dulbecco's Modified Eagle Medium (DMEM; Life Technologies, Grand Island, NY 14072, USA) supplemented with 1 mM sodium pyruvate, 100 mM non-essential amino acids, 25 mM HEPES, 2 mM L-glutamine, 100 U/mL, penicillin, 100 mg/mL streptomycin, and 0.25 mg/mL Fungizone (all from Thermo Fisher Scientific, Grand Island, NY 14072, USA), along with 10% fetal bovine serum (Hyclone, Logan, UT 84321, USA), hereafter referred to as complete DMEM, and cells were enumerated with their viability assessed using the exclusion dye trypan blue (Life Technologies, Grand Island, NY 14072, USA). Cell viability was typically >90%. PBMC isolation after Ficoll-Paque centrifugation was confirmed using a BD FACScan flow cytometer (Becton Dickinson, Franklin Lakes, NJ 07417, USA) using forward scatter (relative cell size) and side scatter (relative cell complexity) settings to assess the proportion of PBMCs in the sample.

### Immunophenotyping

Table 1 lists the primary antibodies, as well as the isotype control antibodies, tested on bottlenose dolphin PBMCs. Human, ovine, and bovine PBMCs were used as positive controls to assure that expected labeling was observed in the species against which the antibodies were raised. For CD4 labeling, 1 × 10^6^ PBMCs were labeled with 100 μl of either fluorochrome-conjugated or unconjugated primary antibodies, as well as with the isotype control antibodies (conjugated or unconjugated), at the dilutions listed in Table 1, for 30 min at 4°C in the dark. PBMCs were then washed with 1 ml PBS and centrifuged at 400 g for 5 min. For fluorochrome-conjugated primary antibodies, PBMCs were then re-suspended with 200 μl of 1% neutral buffered formalin in PBS. For unconjugated antibodies, PBMCs were re-suspended with the 100 μl of a goat anti-mouse FITC conjugated secondary antibody (Life Technologies, Grand Island, NY 14072, USA) for 30 min at 4°C in the dark. PBMCs were then washed with 1 ml PBS, centrifuged, and re-suspended in 200 μl of 1% neutral buffered formalin in PBS.

**Table 1:** Antibodies tested on bottlenose dolphin mononuclear cells.

**Primary antibody (clone)**	**Vendor**	**Documented species reactivity**	**Species tested as positive control**	**Isotype**	**Antibody dilution**
RPA-T4 (CD4)	Thermo Fisher Scientific	Human	Human	IgG1	1:50
VIT4 (CD4)	Miltenyi	Human	Human	IgG2a	1:11
TR-204-33 (CD4)	Tracy Romano	Dolphin	Dolphin	IgG1	1:124
SIM.4 (CD4)	NIH-AIDS	Human	Human	IgG1k	1:100
CACT108A (CD25)	Kingfisher Biotech	Bovine	Bovine	IgG2a	1:100
CACT116A (CD25)	Kingfisher Biotech	Bison, bovine, caprine, ovine, water buffalo	Bovine	IgG1	1:100
MEM-181 (CD25)	Life Technologies	Human, mouse	Human	IgG1	1:100
MEM-140 (CD25)	Life Technologies	Human	Human	IgM	1:100
HI25a (CD25)	Abbiotic	Human	Human	IgG1	1:100
PC61.5-PE (CD25)	Life Technologies	Human, mouse	Mouse	IgG1	1:1,600
9.14 (CD25)	Bio-Rad	Ovine	Ovine	IgG1	1:100
BC96 (CD25)	eBioscience	Human	Human	IgG1	1:5
4E3 (CD25)	Miltyeni	Human, rhesus monkey, cynomolgus monkey	Human	IgG2b	1:50
FJK-16s (FOXP3)	Life Technologies	Bovine, dog, cat, mouse, pig, rat	Dolphin	IgG2a	1:20
Mouse IgG (isotype control)	Life Technologies	n/a	n/a	IgG	1:100
Mouse IgG1 FITC (isotype control)	Life Technologies	n/a	n/a	IgG1	1:5
Mouse IgG2a FITC (isotype control)	Miltyeni	n/a	n/a	IgG2a	1:11
Mouse IgG2b (isotype control)	Miltyeni	n/a	n/a	IgG2b	1:50
Rat IgG1k PE (isotype control)	Life Technologies	n/a	n/a	IgG1	1:1,600
Rat IgG2a kappa (isotype control)	Life Technologies	n/a	n/a	IgG2a	1:20

For CD25 labeling, PBMCs (2 × 10^5^ cells/well) were plated in 96 well flat bottom plates (Fisher Scientific, Agawam, MA 01001, USA) with either no mitogen (complete DMEM alone) or the T cell mitogen, concanavalin A (ConA) at 1 μg/ml. PBMCs were incubated for 24 h at 37°C and 5% CO_2_. Cells were pooled from five replicate wells (for a total of ~1 × 10^6^ cells) in a 5 ml conical tube. Tubes were centrifuged and the cells were re-suspended in 100 μl of either fluorochrome-conjugated or unconjugated primary antibodies, as well as with the isotype control antibodies (conjugated or unconjugated), at the dilutions listed in Table 1, for 30 min at 4°C in the dark. Afterwards, the procedures were the same as with CD4 labeling described above.

The fluorescence of approximately 10,000 lymphocytes was read using a FACScan flow cytometer (Becton Dickinson, Franklin Lakes, NJ 07417, USA) and the automated CellQuest software (Becton Dickinson Immunocytometry System, San Jose, CA 95131, USA). Lymphocytes were identified by their relative size (forward-scattered light, FSC) and their complexity (side-scattered light, SCC). The percent of cells that were positive for CD4 or CD25 were compared to the isotype control for CD4 or CD25. Cells were considered positive for CD4 or CD25 if cell fluorescence was above the isotype control.

For intracellular FOXP3 labeling, 1 × 10^6^ PBMCs were incubated with 100 μl of a 1:100 dilution of the CD4 antibody, SIM.4, for 30 min at 4°C in the dark. Cells were then washed with 1 ml of eBioscience^TM^ flow cytometry staining buffer (Life Technologies, Grand Island, NY 14072, USA) and centrifuged. PBMCs were then re-suspended with 100 μl of a goat anti-mouse FITC conjugated secondary antibody (1:100) (Life Technologies, Grand Island, NY 14072, USA) for 30 min at 4°C in the dark and washed with eBioscience^TM^ staining buffer. Intracellular FOXP3 was labeled with FOXP3 Monoclonal Antibody (FJK-16s), APC, and the Foxp3/Transcription Factor Staining Buffer Set Kit (both from Life Technologies, Grand Island, NY 14072, USA), per Thermo Fisher Scientific's Protocol B: one-step protocol (intracellular (nuclear) proteins) instructions. For the isotype control, an APC labeled rat IgG2a kappa isotype control antibody (Life Technologies, Grand Island, NY 14072, USA) was used at the same recommended dilution as for the FoxP3 antibody (1:20).

The fluorescence of approximately 10,000 lymphocytes was read using a BD Biosciences LSRFortessa X-20 Cell Analyzer (Becton Dickinson, Franklin Lakes, NJ 07417, USA) and FACSDiva software (Becton Dickinson Immunocytometry System, San Jose, CA 95131, USA). Lymphocytes were identified by their relative size (forward-scattered light, FSC) and their complexity (side-scattered light, SCC). Two-color flow cytometry was used to identify cells that were positive for both CD4 and FOXP3. The percent of cells that were positive for FOXP3 was compared to the isotype control for FOXP3.

### Cytokines

Dolphin serum cytokines were quantified using the Bio-Plex Pro^TM^ Human Cytokine Th1/Th2 Panel (Bio-Rad, Hercules, CA 94547, USA) and the Millipore Porcine 3-plex Panel (Millipore, Billerica, MA 01821, USA), as previously described (3). The Th1/Th2 cytokine kit included antibodies to detect IL-2, IL-4, IL-5, IL-10, IL-12, IL-13, IFNγ, TNFα, and GM-CSF. The porcine kit included antibodies to IL-1b, IL-4, and IL-8.

TGFß was quantified using the TGF beta-1 Porcine ProcartaPlex™ Simplex Kit (Thermo Fisher Scientific, Grand Island, NY 14072, USA) or the Bio-Rad Bio-Plex Pro^TM^ TGFß Kit (Bio-Rad, Hercules, CA 94547, USA), according to the manufacturers' instruction. To validate this kit, dolphin and porcine PBMCs were adjusted to 2 × 10^6^ cells/ml and plated in 96 well flat bottom plates, in triplicate wells (2 × 10^5^ cells per well). PBMCs were incubated at 37°C with 5% CO_2_ for a total of 48 h with purified *Escherichia coli* 0111:B4 LPS (lipopolysaccharide; Sigma, St. Louis, MO 63118, USA), a mitogen shown to induce secretion of TGFß (15), at a final concentration of 0.1 μg/ml in pigs, a concentration (referred to as suboptimal for graphic display) previously demonstrated to induce cytokine secretion in humans (16) and pigs (17), and 0.05 or 5.0 μg/ml, which induced suboptimal and optimal B lymphocyte proliferation in bottlenose dolphins (3) and bracketed the concentration used for pigs to maximize the likelihood of detecting a response if one existed. In a separate set of wells, PBMCs were incubated with medium alone, i.e., unstimulated cells, to serve as negative control. At the end of 48 h, tissue culture supernatant was harvested and stored in multiple aliquots at −80°C until analysis.

After the incubation and conjugation process, the plates were measured on the Bio-Plex 200 system (Bio-Rad, Hercules, CA 94547, USA), and analyzed using Bio-Rad Manager 5.0. The observed concentration (pg/ml or ng/ml) of each analyte for each sample was calculated using a curve fit generated for each analyte from seven or eight standards (depending on kit). If a sample concentration was extrapolated outside the standard curve and designated as “Value extrapolated beyond standard range” by the software, that sample concentration was accepted as the calculated value. If a sample concentration was reported as “out of range” by the software, that sample concentration was given a 0 pg/ml or 0 ng/ml value or the highest value on the standard curve, depending on whether it was below or above the measurable range.

Prior to each use of the Bio-Plex 200 system, an instrument calibration and validation procedure using the Bio-Rad Validation and Calibration kit (Bio-Rad, Hercules, CA 94547, USA) was performed to assure the instrument was performing properly, as per manufacturer's instruction. The instrument passed both calibration and validation tests prior to each use.

### Lymphocyte Stimulation With Th1, Th2, or Treg Cytokines

In order to assess ability of dolphin lymphocytes to respond to a Th1, Th2, or Treg stimulus, dolphin PBMCs (2 × 10^6^ cells/ml) were incubated in 96 well flat bottom plates with human recombinant cytokines at concentrations of 0 (unstimulated), 1, 10, and 25 pg/ml for 24 h. To assess a Th1 response, cells were stimulated with IL-12 (Millipore Sigma, Burlington, MA 01803, USA) and IFNγ (Thermo Fisher Scientific, Grand Island, NY 14072, USA) and analyzed for IFNγ expression. To stimulate a Th2 response, cells were stimulated with IL-4 (Millipore Sigma, Burlington, MA 01803, USA) and analyzed for IL-4 and IL-13 expression. To assess a Treg response, cells were stimulated with IL-2 (Thermo Fisher Scientific, Grand Island, NY 14072, USA) and TGFβ (Thermo Fisher Scientific, Grand Island, NY 14072, USA) and analyzed for TGFβ and IL-10 expression. After a 24 h incubation at 37°C and 5% CO_2_, cells were collected from the plates, centrifuged at 220 g for 10 min, re-suspended in RNAlater solution (Thermo Fisher Scientific, Grand Island, NY 14072, USA) and stored at 4°C for up to 1 month. RNAlater samples were then moved to −20°C for long-term storage.

### Primers

Primers were chosen based on published bottlenose dolphin primer sequences (18, 19). Primer sequences are reported in Table 2.

**Table 2:** Sequence of the forward and reverse primers used to amplify housekeeping and cytokine genes.

**Cytokine**		**Sequence: 5′ to 3′**	**References**
S-9	Forward	GAGGATTTCTTGGAGAGACGCCTG	(18, 19)
	Reverse	CTTGCGGACCCTGATATGGCGC	
HPRT1	Forward	GTGGCCCTCTGTGTGCTC	(19)
	Reverse	ACTATTTCTGTTCAGTGCTTTGATGT	
IFNγ	Forward	CAGAGCCAAATAGTCTCCTTCTACTT	(18)
	Reverse	CTGGATCTGCAGATCATCTACCGGAATTTG	
IL-4	Forward	GGAGCTGCCTGTAGAAGACGTCTTTG	(18)
	Reverse	CTTCATTCACAGAACAGGTCATGTTTGCC	
IL-13	Forward	CCTCTACAGCCCTCAAGGAGC	(18)
	Reverse	CTTCCAGGGCTGAACAGTACATGT	
TGFß	Forward	GAGCTGCGCCTGCTGAGGCT	(18)
	Reverse	CCTCTATTTCCTCTCCGTGGGTC	
IL-10	Forward	GACTTTAAGGGTTACCTGGGTTG	(18)
	Reverse	TCCACTGCTTTGCTCTTGTTTTC	

### Gene Expression

RNA was extracted from dolphin PBMCs samples using a RNeasy Mini Kit (Qiagen, Valencia, CA 91355, USA), and genomic DNA was removed using a TURBO DNA-free kit (Thermo Fisher Scientific, Grand Island, NY 14072, USA). RNA concentration was determined using a Qubit fluorometer (Thermo Fisher Scientific, Grand Island, NY 14072, USA). After isolation and quantification, RNA was reverse transcribed into cDNA with 100 ng RNA per reaction using a high capacity cDNA reverse transcription kit (Thermo Fisher Scientific, Grand Island, NY 14072, USA). Real time PCR (qRT-PCR) reactions were performed using SYBR green (Thermo Fisher Scientific, Grand Island, NY 14072, USA) on a CFX96 Real-Time PCR Detection System (Bio-Rad, Hercules, CA 94547, USA). All samples were analyzed for housekeeping genes HPRT1 and S-9. Cycling conditions for genes were 95°C for 10 min, 40 cycles of denaturation at 95°C for 15 s and annealing at 63°C for 1 min, followed by a dissociation stage. For IL-4 and IL-10, the annealing temperature was 54°C. Reactions containing water, but no cDNA, were used as negative controls. Product specificity was monitored by analysis of melting curves. Gene expression data were analyzed using the Comparative C_T_ (ΔΔC_T_) Method. Samples for which the amplification of the housekeeping genes were outside of the expected range were discarded, so as to not misinterpret a change in the expression of a target gene as an inadequate PCR reaction.

### Mitogen-Induced T Lymphocyte Proliferation

Lymphocyte proliferation was evaluated as previously described (3). Briefly, lymphocytes were incubated with mitogens for 66 h in flat-bottom 96-well plates (Fisher Scientific, Agawam, MA 01001, USA) at 37°C and 5% CO_2_. Mitogens chosen included two T cell mitogens (ConA and phytohemagglutinnin A, or PHA). Mitogens were used at optimal as well as suboptimal concentrations, since suboptimal concentrations of mitogens allowed for higher sensitivity to subtle deficits when optimal concentrations of mitogens did not reveal differences (20). Lymphocyte proliferation was evaluated by the incorporation of 5-bromo-2′-deoxyuridine (BrdU), a thymidine analog, detected with a monoclonal antibody and colorimetric enzymatic reaction (Cell Proliferation ELISA BrdU (colorimetric), Roche Diagnostics GmbH, Mannheim, Germany) as per manufacturer's instructions using an ELISA plate reader (Multiskan EX v.1.0) at 450 nm with a reference wavelength of 690 nm. Results were expressed as optical density (OD).

Cells from mice (one mouse for each experimental day) were assayed concurrently with dolphin samples for quality control, as previously described (2). After field sampling, mouse data were assessed for the presence of outliers using the SPSS software (IBM SPSS Statistics version 21, Armonk, NY 10504, USA). If outliers were detected, it was assumed that normal daily variability for the assay was exceeded, and the corresponding dolphin data for that assay on that day were eliminated from the dataset.

### Statistical Analyses

Correlation and regression analyses as well as *t*-tests were conducted using the SPSS software (IBM SPSS Statistics version 21, Armonk, NY 10504, USA) and Principal Component Analysis was performed using the Minitab software v. 18.1 (Minitab, Inc., State College, PA 16801, USA).

## Results

**Figure 1:**
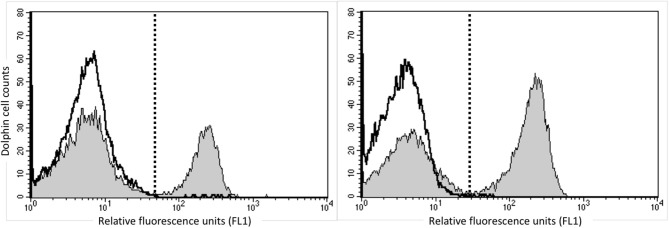
Identification of CD4+ bottlenose dolphin cells using flow cytometry. Dolphin PBMCs were labeled with the commercially available anti-human CD4 SIM.4 (left) or research anti-cetacean CD4 TR202 (right). The white histogram represents isotype control and the gray histogram represents CD4 labeling, and the dashed line discriminates positive (right) from negative (left) labeling, with isotype control consistently including <1% background. Both antibodies labeled ~30% of bottlenose dolphin peripheral blood lymphocytes.

**Figure 2:**
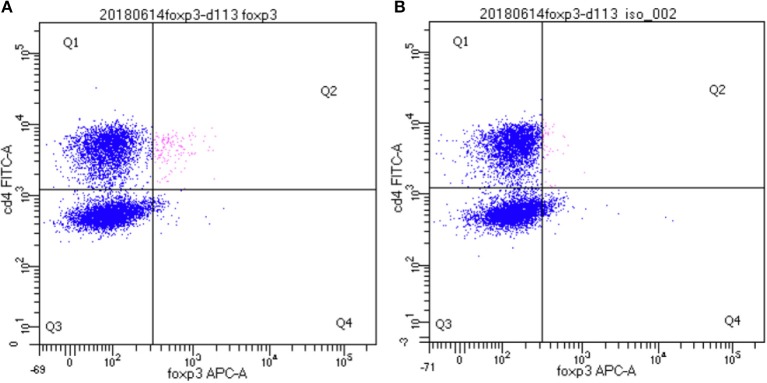
Identification of CD4+/FOXP3+ bottlenose dolphin Treg cells using flow cytometry. A distinct population of CD4+/FOXP3+ cells are observed in the upper right quadrant (Q2, in pink) **(A)** that was absent upon labeling with isotype control for FOXP3 **(B)**.

In order to identify dolphin Treg cells, we tested a battery of antibodies to CD4, CD25, and FOXP3 (Table 1). Immunolabeling was observed for all antibodies in the species of origin (human, bovine, and ovine) used as a positive control (data not shown). A cross-reacting antibody to human CD4 (SIM.4) labeled on average 32% (SD = 8%) of bottlenose dolphin lymphocytes (*n* = 20), while an antibody generated against cetacean CD4 (TR202) labeled 23 and 36% of bottlenose dolphin peripheral blood lymphocytes in preliminary experiments (*n* = 2), when the fluorescence of isotype control was subtracted from that of cells labeled with a CD4 antibody (Figure 1), while three other antibodies to human CD4 did not cross-react. None of the nine antibodies to CD25 tested cross-reacted with dolphin lymphocytes, with or without ConA stimulation to induce expression as observed in positive control species (data not shown). The antibody to FOXP3 clearly labeled a distinct population of CD4+ lymphocytes not present in cells labeled with the isotype control (Figure 2). In Sarasota Bay dolphins, when isotype control was subtracted, 0.67 ± 0.40% of all dolphin lymphocytes were FOXP3+, and 1.93 ± 1.07% of dolphin CD4+ lymphocytes were FOXP3+. The proportions of lymphocytes were FOXP3+ and of dolphin CD4+ lymphocytes were FOXP3+ were not significantly different between males and females, and were not significantly correlated with age (data not shown).

**Figure 3:**
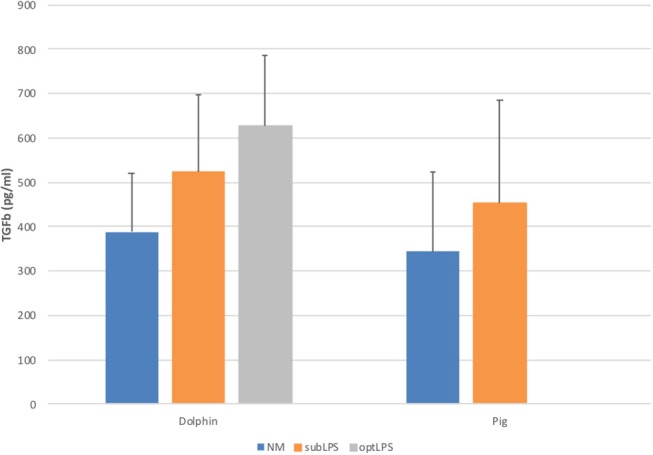
Tissue culture supernatant concentrations of TGFß following a 48 h stimulation of porcine (*n* = 3) or dolphin (*n* = 5) PBMCs with LPS at sub-optimal (subLPS, 0.05 μg/ml for dolphin and 0.1 μg/ml for pigs) and optimal (optLPS, 5.0 μg/ml for dolphin) concentrations, compared to no mitogen stimulation (NM). TGFß concentrations were measured using the Luminex platform with porcine reagents. Data are presented as mean and error bars represent standard error of the mean.

In order to determine the functionality of dolphin Treg cells, we validated the use of human and porcine reagents to measure TGFß, the major Treg effector cytokine. To do so, we stimulated dolphin and porcine PBMCs with LPS *in vitro* to induce the secretion of TGFß in the tissue culture supernatant. Dolphin lymphocytes showed a concentration-response increase in TGFß production upon stimulation with LPS using the porcine reagents (34 and 61% increase in TGFß production with 0.05 and 5.0 μg/ml of LPS, respectively), as did porcine lymphocytes (31% increase in TGFß production with sub-optimal concentration, Figure 3). The use of human reagents did not allow the measurement of such an increase in the expression of TGFß in dolphin cells upon stimulation with LPS (data not shown). We therefore concluded that the porcine, but not the human reagents, cross-reacted in a specific manner with dolphin TGFß, and further experiments were performed using the porcine reagents.

**Figure 4:**
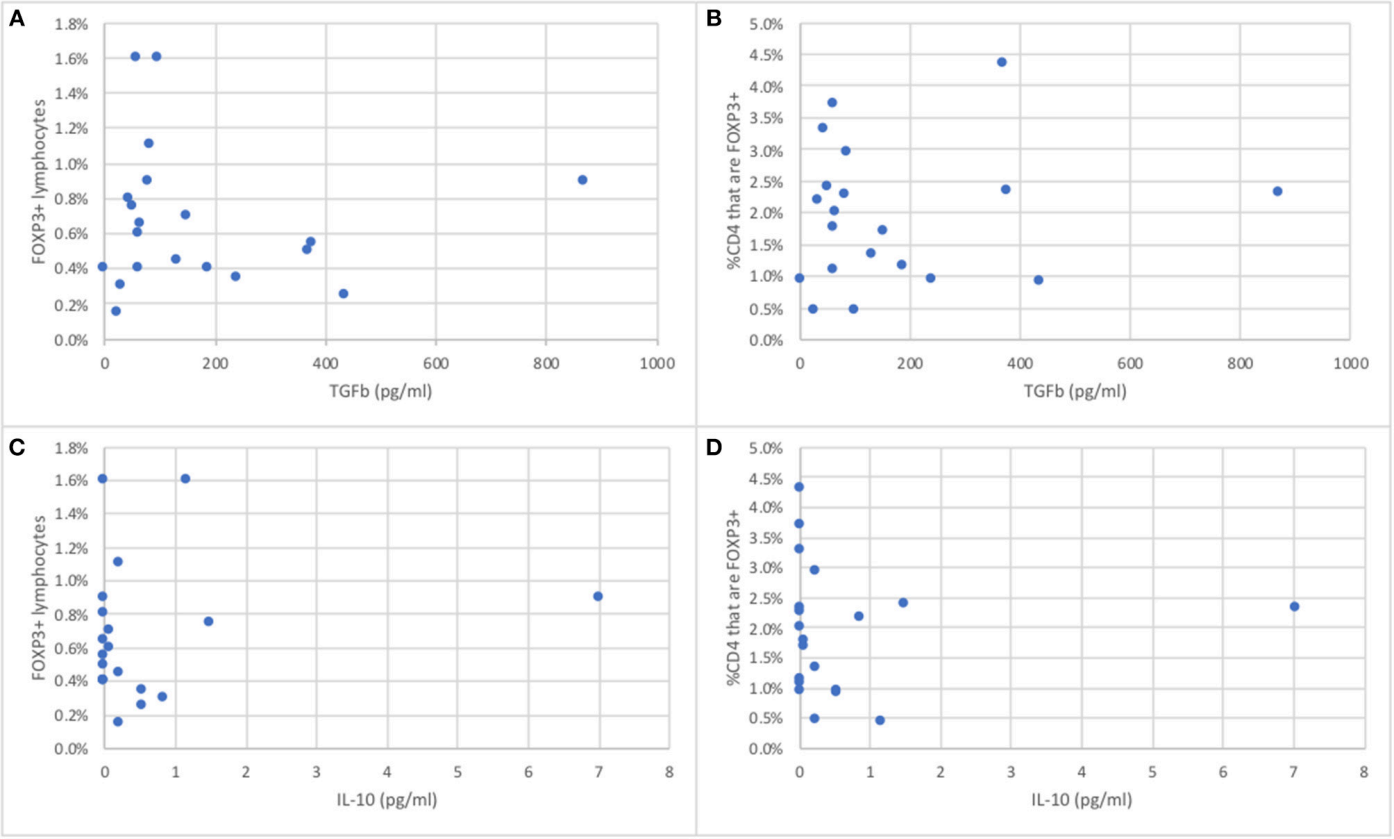
Relationship between the proportion of peripheral blood FOXP3+ dolphin lymphocytes **(A,C)** or the proportion of CD4+ T cells that are FOXP3+ **(B,D)** and serum concentrations of TGFß **(A,B)** or IL-10 **(C,D)**, the major Treg effector cytokines (*n* = 20).

The serum concentrations of TGFß in 20 wild bottlenose dolphins from Sarasota Bay ranged from 0 to 868 pg/ml, with an average of 170 pg/ml and a standard deviation of 106, while IL-10 ranged from 0 to 7 pg/ml, with an average of 0.62 pg/ml and a standard deviation of 1.01. The serum concentrations of TGFß were not significantly different between males and females, and were not significantly correlated with age (data not shown). In order to assess if the proportion of peripheral blood FOXP3+ dolphin lymphocytes were representative of Treg function we tested the hypothesis that dolphins with more FOXP3+ lymphocytes had higher levels of serum TGFß and IL-10, the two major Treg effector cytokines. There was no significant correlation between either dolphin serum TGFß or IL-10 concentrations and the proportion of FOXP3+ lymphocytes, or with the proportion of CD4+ lymphocytes that were FOXP3+ (Figure 4).

**Figure 5:**
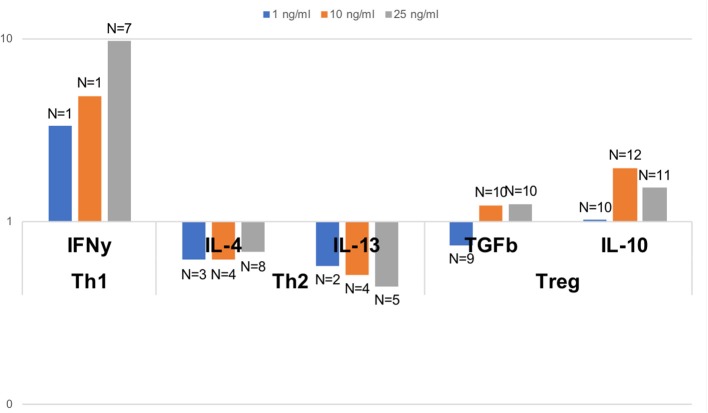
Relative gene expression of effector Th1, Th2, and Treg cytokines upon polarizing stimulus with the Th1 inducing cytokines IL-2 and IFNγ, the Th2 inducing cytokine IL-4, and the Treg inducing cytokines IL-2 and TGFß, all tested at 1, 10, and 25 pg/ml. Data are expressed relative to unstimulated cells (value of 1). Dolphin PBMCs responded most robustly to a Th1 stimulus, and to some extent to a Treg stimulus (at some but not all concentrations), but Th2 polarization with IL-4 resulted in a down-regulation, and not an enhancement, of the expression of the genes for IL-4 and IL-13. The number of dolphins tested for each stimulation condition, once samples with inadequate expression of housekeeping genes were removed, is indicated on the figure over the bars.

In order to assess the capacity of dolphin Th cell subsets to respond to a Th1, Th2, or Treg stimulus, we tested the relative expression of effector cytokines upon *in vitro* stimulation with human recombinant cytokines, as previously done on a limited basis in cetaceans (21, 22). A robust 9.7 fold increase in the expression of the gene for IFNγ was observed upon stimulation with 25 pg/ml IL-12 and IFNγ compared to unstimulated cells, with a clear dose-response pattern (Figure 5). An increase in Th2 cytokines was not observed upon stimulation with 25 pg/ml IL-4; rather, IL-4 down-regulated the expression of IL-4 and IL-13 to 68 and 44%, respectively, of the levels in unstimulated cells (Figure 5). This down-regulation was clearly dose-dependent for IL-13, but not for IL-4. Treg stimulation by IL-2 and TGFß resulted in nearly no change at 1 pg/ml, a modest up-regulation of the genes for TGFß and IL-10 (1.2 and 2.0 fold increase, respectively, at 10 pg/ml, and 1.3 and 1.5 fold increase, respectively, at 25 pg/ml), compared to unstimulated cells (Figure 5).

**Figure 6:**
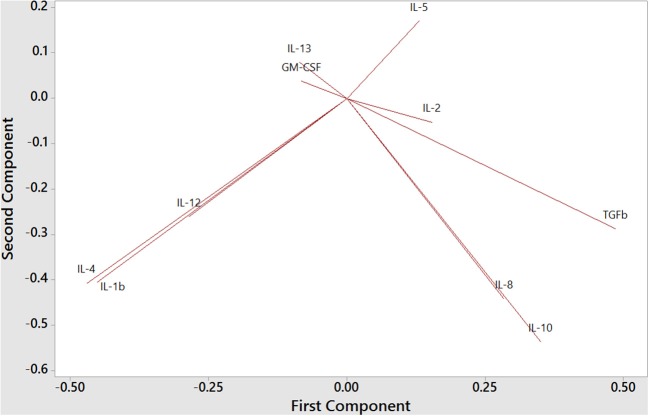
Principal Component Analysis of Th1, Th2, Treg, and inflammatory cytokines in bottlenose dolphin serum (*n* = 20), according to the first two components (first component on the x axis and second component on the y axis).

To understand the balance of cytokines in wild dolphins, we measured serum concentrations of 12 cytokines including the Th1 cytokines IL-2, IL-12, and IFNγ, the Th2 cytokines IL-4, IL-5, and IL-13, the Treg cytokines IL-10 and TGFß, and the inflammatory cytokines IL-1ß, IL-8, TNFα, and GM-CSF, in 20 wild dolphins and performed Principal Component Analysis (PCA) to see how cytokines clustered (Figure 6). The plot of the first two components showed the Treg cytokines IL-10 and TGFß varying in the same general direction, which was also similar to that for IL-8. Further, IL-4 and IL-1ß, and to a lesser extent IL-12, varied in a similar direction, which was different from that for the previous cluster. IL-5 and IL-13 varied in different directions, which were also different from that for IL-4. GM-CSF varied in a direction which was closest to that for IL-13, and IL-2 varied in a direction that was closest to that for TGFß. Note the absence of IFNγ and TNFα that were removed from the analysis by the software as they did not include variance in this dataset.

We further assessed the relationship between serum cytokine concentrations in wild dolphins and the results of *in vitro* mitogen-induced T cell proliferation using regression analysis. The only cytokines that significantly contributed to explaining the variability in T cell proliferation were IL-4 upon sub-optimal and optimal stimulation with ConA and optimal stimulation with PHA, and IL-2, IL-10, and GM-CSF upon sub-optimal stimulation with PHA (Table 3). The *R*^2^ for models explaining the variability of T cell proliferation with IL-4 upon sub-optimal and optimal stimulation with ConA and optimal stimulation with PHA were relatively low, whereas the *R*^2^ for the model explaining the variability T cell proliferation with IL-2, IL-10, and GM-CSF upon sub-optimal concentration of PHA was considerably higher. Further, regression analysis did not find a significant contribution of the proportion of FOXP3+ lymphocytes in explaining the variability of mitogen-induced T lymphocyte proliferation using either sub-optimal or optimal concentrations of the mitogens ConA or PHA (data not shown).

**Table 3:** Forward regression analysis to assess which cytokines contribute significantly to explaining the variability of T lymphocyte proliferation (*n* = 20).

**Mitogen**	**Variables**	**Model *R*^**2**^**	**Model *p*-value**
Sub ConA	IL-4	0.284	0.015
Opt ConA	IL-4	0.310	0.011
Sub PHA	IL-2, IL-10, GM-CSF	0.737	0.008
Opt PHA	IL-4	0.264	0.025

## Discussion

We have, for the first time to the authors' knowledge, detected the presence of Treg cells in bottlenose dolphin blood, and shown the potential for bottlenose dolphins to mount a Th1, Th2, and Treg response. This represents a significant new step in fundamental and applied dolphin immunology, which will help us better understand the importance of these responses in health and disease.

Antibodies were used to label dolphin cell subsets. Studies requiring the use of antibodies in marine mammals are challenging given the paucity of reagents available, and the lack of commercially available marine mammal specific antibodies. In addition to a small number of antibodies developed against marine mammal leukocyte subset markers (23–29), several studies reported the use of cross-reactive monoclonal antibodies to label leukocyte subsets in marine mammals (30–38). This study used blood samples from original target species or species with documented cross reactivity as positive control, to assure quality control in documenting lack of cross-reactivity with dolphin cells.

We successfully detected a subset of CD4+ lymphocytes in dolphin blood samples. The specificity of SIM.4 for labeling beluga whale CD4 has previously been demonstrated through immunoprecipitation of a 56.4 kDa protein in beluga, in the range of the 53.5 kDa protein in human (30), and the proportion of lymphocytes labeled in bottlenose dolphins (*X* = 32.2%, *SD* = 8.3%) was similar to that previously reported in belugas (*X* = 29.8%, *SD* = 6.9%). This is not surprising since bottlenose dolphins and beluga whales CD4 molecules share 98% homology at the nucleotide level and 97% at the amino acid level (39). In addition, the proportion of CD4+ cells over the sum of CD2+ T lymphocytes and CD19+ B lymphocytes in “normal” dolphins was also 30% in a field study to quantify the immune changes in bottlenose dolphins with lobomycosis (26). Further, in this study, SIM.4 labeled a relatively similar proportion of lymphocytes as TR202, an anti-CD4 developed specifically against bottlenose dolphin CD4 (26, 27).

This study did not successfully identify CD25+ dolphin lymphocytes, despite the use of unstimulated and stimulated T cells to increase the frequency of those cells. No study has reported cross-reactive antibodies to marine mammal CD25, except for one study that tested one antibody in dolphins and stated that it cross-reacted but was not selected for further use because of “low detectability” without further explanation (40). A separate study also reported the lack of cross-reactivity in dolphin of two antibodies to CD25 (38), although they did not appear to test upon stimulation as we did. An earlier study used fluorochrome-labeled IL-2 to detect the increase in surface expression of IL-2 receptor on the surface of mitogen-activated bottlenose dolphin lymphocytes (41), however this reagent unfortunately appears to be no longer available commercially.

This study successfully identified FOXP3+ dolphin lymphocytes. Treg cells are generally defined as CD4+/CD25+/FOXP3+ lymphocytes (11). While we could not find a cross-reactive antibody to CD25, we observed the presence of a clear and distinct population of CD4+/FOXP3+ Treg cells in dolphin blood samples. The antibody to FOXP3 used in this study appeared to be broadly cross-reactive, with demonstrated labeling in cows (42), sheep (43), pigs (44), and dogs (45), in addition to the numerous species cited by the manufacturer, including cats, mice and rats (eBioscience/ThermoFisher Scientific). Dolphin CD4+/FOXP3+ Treg cells generally represented <1% of lymphocytes, a proportions very similar to what was observed in cows (~0.66%) (42), sheep (4.6%), (43), pigs (~1.6%) (44), and dogs (1–2.6%) (45). The relatively small proportion of Treg cells labeled in this study is not a concern given the sensitivity and precision of the instrument used and the consistent specificity of the labeling (Figure 2). While we have not ruled out the possibility that some of the CD4+/FOXP3+ dolphin cells identified as Treg in this study are CD25-, the literature in other species consistently reports the vast majority of CD4+/FOXP3+ cells are CD25+ (42–45). Overall, the labeling pattern limited to CD4+ cells in dolphins, the proportion of PBMCs labeled, and the specificity of FOXP3 labeling for Treg cells (42, 44), altogether suggest the specificity of FJK-16s labeling for dolphin Treg cells.

Cytokines are important mediators of the immune response, and several studies have explored the use of cytokines to relate to health status of marine mammals in different manners. Amplification of mRNA for cytokines using quantitative reverse-transcription polymerase chain reaction (qRT-PCR) to quantify cytokine expression in marine mammals, with or without stimulation of PBMCs, has been used in several studies (18, 46–53). While useful to measure responsiveness to a signal, the quantification of mRNA does not necessarily represent the circulating bioactive protein, which requires important steps, in which translation and secretion need to take place. One study used brefeldin to block secretion and flow cytometry with intra-cellular labeling for cytokines using cross-reactive monoclonal antibodies (54). While powerful at associating cytokine production with specific cell types using simultaneous extra-cellular labeling, this approach is tedious and time consuming, and allows the labeling for a relatively low number of cytokines at a time, in this case only two cytokines. Similarly, immunohistochemistry has been used to detect cytokines in cetacean tissue section (53, 55, 56). While informative on the distribution of the cell types secreting cytokines in different tissues, this method is limited to the use of tissues from dead animals, or biopsies in live animals, which would be a rather invasive procedure. ELISAs have been developed to measure killer whale-specific IL-6 (57) and dolphin-specific IFNγ and TNFα (58) in serum and tissue culture medium. While sensitive and reproducible under stringent laboratory conditions, ELISAs are relatively time consuming and labor-intensive. This study focused on measuring bioactive cytokines circulating in serum using the relatively high-throughput Luminex technology and highly standardized commercial reagents for highly replicable results (3, 59). With the specificity of human and porcine reagents was validated based on the detection of the analytes in dolphin serum and *in vitro* cell responsiveness to stimuli similar to the species of origin (59), this new approach might significantly advance the use of cytokine measurements as part of marine mammal health assessment.

While bottlenose dolphin Treg cells could be identified and quantified, we also wanted to assess their functionality by measuring their effector cytokines. Our lab previously validated the use of human reagents to detect bottlenose dolphin IL-10 using the Luminex platform and human Th1/Th2 reagent kits (3). The present study validated the use of porcine, but not human reagents, to document a <2 fold increase in expression of TGFß upon *in vitro* stimulation with LPS, as seen using porcine PBMCs (Figure 3) and goat macrophages (15). This validation allowed the quantification of TGFß in the serum of free-ranging bottlenose dolphins, as a reference for future comparisons.

The evaluation of Treg should include their identification as well as their functions. Our inability to label CD25 on dolphin cells prevented us from sorting live CD4+ CD25^hi^ CD127^low^ Tregs for functional assays, as described by others (60). Intracellular labeling for FOXP3 required cell fixation and permeabilization, and cells could no longer be used in functional assays. However, we could induce the expression of the gene for TGFß and IL-10 upon stimulation with TGFß and IL-2, as expected for functional Tregs (11), demonstrating the functionality of Treg in bottlenose dolphins. Importantly, the clustering of serum IL-10 and TGFß using PCA in our subset of dolphins suggested the potential for actual *in vivo* Treg polarization and differentiation. The proportion of circulating Treg lymphocytes was not correlated with serum concentrations of the Treg effector cytokines TGFß and IL-10, or with mitogen-induced T lymphocyte proliferation, suggesting that not all circulating Treg cells are functional and active. This is not surprising given the demonstration that the proliferation (expansion of the pool) of Treg and their functional suppressive capacity are driven by different pathways (61). Further, while the effector T cell subsets Th1, Th2, and Th17 mostly draw their energy through glycolysis, Treg use fatty acid oxidative pathways for energy (62), and it is possible that qualitative or quantitative differences in serum lipids may affect the functioning of Treg cells in dolphins. With the diversity of emerging receptors and pathways involved in the modulation of Treg cells (63), it is also possible that the proliferation or functions of dolphin Treg may be affected by environmental contaminants such as dioxin-like PCBs via the Ah receptor. It is also understood that the regulatory functions of Treg cells are not solely modulated by their effector cytokines, and in part require cell-cell interactions (6, 64). The proportion of circulating FOXP3+ lymphocytes did not contribute to significantly explaining *in vitro* mitogen-induced T cell proliferation in our study, which may relate to the relatively small number of dolphins used and/or the use of mitogens that stimulate T cell proliferation in a manner that likely exceeds physiological stimuli. It would be valuable to find reagents to sort bottlenose dolphin live Treg cells for use in functional test for suppressive activity as described in humans (60). Additional studies are required to further characterize the functionality of Treg cells in bottlenose dolphins.

There is mounting evidence for the importance of the Th1/Th2 balance and functionality to appropriately respond to immunological challenges. While Th1 and Th2 cells have not been specifically identified or quantified in this study, we demonstrated a robust expression of the gene for IFNγ upon stimulation with IL-12 and IFNγ, as expected for Th1 cells (11), suggesting the potential for Th1 polarization and differentiation in bottlenose dolphin Th cells. Stimulation with IL-4, however, unexpectedly did not induce the expression of the genes for the Th2 cytokines IL-4 and IL-13. This may be due to the requirement for IL-2 for a Th2 polarization as described in mice (65). It is interesting that the Th2 cytokines IL-4, IL-5, and IL-13 did not cluster closely using PCA, suggesting a potential disconnect between the different effector cytokines in the wild bottlenose dolphins sampled. However, IL-4, and to a lesser extent IL-12, clustered closely with the pro-inflammatory cytokine IL-1ß, suggesting the potential ability of both the Th1 and Th2 response to be triggered upon acute inflammatory signals, and the possibility that a Th2 response might be favored. Regression analysis including IL-4 as a significant contributor to explaining the variability in mitogen-induced T lymphocyte proliferation under three out of four scenarios may support a preference for a Th2 over Th1 response in bottlenose dolphin. Continued studies to better understand the relationships between Th1 and Th2 cytokines and immune responsiveness will be critical to better understand the functionality of the immune system in dolphins.

This study focused on live captures of wild dolphins, and the authors acknowledge that the chase and capture, with associated stress response, could have influenced some aspects of the immune functions measured. However, the focus on live captures of wild dolphins will allow direct comparisons with health assessments in other populations of wild dolphins, subjected to challenges such as exposure to environmental contaminants or disease outbreaks, that are also captured using similar methods.

Overall, this study for the first time demonstrated the ability to quantify FOXP3+ Treg cells in bottlenose dolphins, and the potential for polarization and functional differentiation of Th cells toward a Th1 or Treg response. While we have not directly demonstrated the polarization and functional differentiation of Th cells toward a Th2 response, we provided evidence for such potential, and the potential for a preferential Th2 over Th1 response in bottlenose dolphin in a relatively small subset of individuals from a well-studied reference population of bottlenose dolphins. These results may be useful in better understanding the mechanisms by which the dolphin immune system is affected upon exposure to environmental challenges and how it responds to challenges with pathogens.

## Ethics Statement

Dolphin samples were collected under National Marine Fisheries Service Scientific Research Permit No. 20455, issued to RW. This study was carried out in accordance with the recommendations of Institutional Animal Care and Use Committee (IACUC) at Mote Marine Laboratory and at the University of Connecticut. The protocol was approved by the Institutional Animal Care and Use Committee (IACUC) at Mote Marine Laboratory and at the University of Connecticut. Human, bovine, ovine, and porcine blood purchased from commercial sources was deemed exempt from IACUC oversight by the University of Connecticut IACUC.

## Author Contributions

SD and ML are responsible for the study design. SD and RW participated in the dolphin captures and sampling, which was overseen by RW. ML and LJ performed the laboratory work, with advice from GR for experiments on gene expression. SD, ML, LJ, GR, and RW participated in the drafting and editing of the manuscript. All authors contributed to manuscript revision, read and approved the submitted version.

### Conflict of Interest Statement

The authors declare that the research was conducted in the absence of any commercial or financial relationships that could be construed as a potential conflict of interest. The reviewer TR declared a shared affiliation, with no collaboration, with two of the authors, SD and ML, to the handling editor at the time of review. The reviewer TR provided the authors with a monoclonal antibody but was not otherwise involved in this work, nor in any on-going projects with the authors.

## References

[1] Levin M Marine mammal immunology. In: Dierauf L Gulland FMD Whitman K, editors. CRC Handbook of Marine Mammal Medicine: Health, Disease, and Rehabilitation. 3rd ed. Boca Raton, FL: CRC Press (2018, 209–229.

[2] Schwacke LH Zolman ES Balmer BC De Guise S George RC Hoguet J. Anaemia, hypothyroidism and immune suppression associated with polychlorinated biphenyl exposure in bottlenose dolphins (*Tursiops truncatus*). Proc Biol Sci. (2012) 279:48–57. 10.1098/rspb.2011.0665 21613298 PMC3223648

[3] De Guise S Levin M Gebhard E Jasperse L Burdett Hart L Smith CR Changes in immune functions in bottlenose dolphins in the northern Gulf of Mexico associated with the Deepwater Horizon oil spill. Endang Species Res. (2017) 33:291–303. 10.3354/esr00814

[4] Schwacke LH Twiner MJ De Guise S Balmer BC Wells RS Townsend FI. Eosinophilia and biotoxin exposure in bottlenose dolphins (*Tursiops truncatus*) from a coastal area impacted by repeated mortality events. Environ Res. (2010) 110:548–55. 10.1016/j.envres.2010.05.003 20537621

[5] Kuby J Immunology. 3rd ed. New York, NY: W.H. Freeman and Company (1997).

[6] Yagi H Nomura T Nakamura K Yamazaki S Kitawaki T Hori S. Crucial role of FOXP3 in the development and function of human CD25+CD4+ regulatory T cells. Int Immunol. (2004) 16:1643–56. 10.1093/intimm/dxh165 15466453

[7] Aghili B Amirzargar AA Rajab A Rabbani A Sotoudeh A Assadiasl S. Altered suppressor function of regulatory T cells in type 1 diabetes. Iran J Immunol. (2015) 12:240–51. 26714416 10.22034/iji.2015.16753

[8] Beres AJ Drobyski WR. The role of regulatory T cells in the biology of graft versus host disease. Front Immunol. (2013) 4:163. 10.3389/fimmu.2013.00163 23805140 PMC3690651

[9] Ohl K Tenbrock K. Regulatory T cells in systemic lupus erythematosus. Eur J Immunol. (2015) 45:344–55. 10.1002/eji.201344280 25378177

[10] Alzabin S Williams RO. Effector T cells in rheumatoid arthritis: lessons from animal models. FEBS Lett. (2011) 585:3649–59. 10.1016/j.febslet.2011.04.034 21515267

[11] Povoleri GA Scotta C Nova-Lamperti EA John S Lombardi G Afzali B. Thymic versus induced regulatory T cells - who regulates the regulators? Front Immunol. (2013) 4:169. 10.3389/fimmu.2013.00169 23818888 PMC3694260

[12] Yamane H Paul WE. Cytokines of the gamma(c) family control CD4+ T cell differentiation and function. Nat Immunol. (2012) 13:1037–44. 10.1038/ni.2431 23080204 PMC4825860

[13] Schwacke LH Smith CR Townsend FI Wells RS Hart LB Balmer BC Health of common bottlenose dolphins (*Tursiops truncatus*) in Barataria Bay, Louisiana, following the Deepwater Horizon oil spill. Environ Sci Technol. (2014) 48:93–103. 10.1021/es403610f 24350796

[14] Wells RS Rhinehart HL Hansen LJ Sweeney JC Townsend FI Stone R Bottlenose dolphins as marine ecosystem sentinels: developing a health monitoring system. EcoHealth. (2004) 1:246–54. 10.1007/s10393-004-0094-6

[15] Walia V Kumar R Mitra A. Lipopolysaccharide and concanavalin A differentially induce the expression of immune response genes in caprine monocyte derived macrophages. Anim Biotechnol. (2015) 26:298–303. 10.1080/10495398.2015.1013112 26158463

[16] Eggesbo JB Hjermann I Lund PK Joo GB Ovstebo R Kierulf P LPS-induced release of IL-1 beta, IL-6, IL-8, TNF-alpha and sCD14 in whole blood and PBMC from persons with high or low levels of HDL-lipoprotein. Cytokine. (1994) 6:521–9. 10.1016/1043-4666(94)90080-9 7530060

[17] Levin M Jasperse L Gebhard E Rousselet E Walsh C. Lack of cross-reactivity of human and porcine reagents to quantify manatee (*Trichechus manatus*) cytokines. Vet Immunol Immunopathol. (2018) 203:57–9. 10.1016/j.vetimm.2018.07.012 30243374

[18] Sitt T Bowen L Blanchard MT Smith BR Gershwin LJ Byrne BA. Quantitation of leukocyte gene expression in cetaceans. Dev Comp Immunol. (2008) 32:1253–9. 10.1016/j.dci.2008.05.001 18572242

[19] Chen IH Chou LS Chou SJ Wang JH Stott J Blanchard M. Selection of suitable reference genes for normalization of quantitative RT-PCR in peripheral blood samples of bottlenose dolphins (*Tursiops truncatus*). Sci Rep. (2015) 5:15425. 10.1038/srep15425 26486099 PMC4614023

[20] Mori C Morsey B Levin M Nambiar PR De Guise S. Immunomodulatory effects of *in vitro* exposure to organochlorines on T-cell proliferation in marine mammals and mice. J Toxicol Environ Health. (2006) 69:283–302. 10.1080/15287390500227472 16407088

[21] De Guise S Ross PS Osterhaus AD Martineau D Beland P Fournier M. Immune functions in beluga whales (*Delphinapterus leucas*): evaluation of natural killer cell activity. Vet Immunol Immunopathol. (1997) 58:345–54. 10.1016/S0165-2427(97)00035-4 9436277

[22] De Guise S Bernier J Dufresne M Martineau D Beland P Fournier M Immune functions in beluga whales (*Delphinapterus leucas*): evaluation of mitogen-induced blastic transformation of lymphocytes from peripheral blood, spleen and thymus. Vet Immunol Immunopathol. (1996) 50:117–26. 10.1016/0165-2427(95)05490-1 9157677

[23] De Guise S Erickson K Blanchard M Dimolfetto L Lepper H Wang J. Characterization of a monoclonal antibody that recognizes a lymphocyte surface antigen for the cetacean homologue to CD45R. Immunology. (1998) 94:207–12. 10.1046/j.1365-2567.1998.00483.x 9741342 PMC1364206

[24] De Guise S Erickson K Blanchard M DiMolfetto L Lepper HD Wang J. Monoclonal antibodies to lymphocyte surface antigens for cetacean homologues to CD2, CD19 and CD21. Vet Immunol Immunopathol. (2002) 84:209–21. 10.1016/S0165-2427(01)00409-3 11777535

[25] De Guise S Erickson K Blanchard M DiMolfetto L Lepper HD Stott JL. Characterization of F21.A, a monoclonal antibody that recognize a leucocyte surface antigens for killer whale homologue to ß-2 integrin. Vet Immunol Immunopathol. (2004) 97:195–206. 10.1016/j.vetimm.2003.09.006 14741138

[26] Reif JS Peden-Adams MM Romano TA Rice CD Fair PA Bossart GD. Immune dysfunction in Atlantic bottlenose dolphins (*Tursiops truncatus*) with lobomycosis. Med Mycol. (2009) 47:125–35. 10.1080/13693780802178493 18608890

[27] Romano TA Ridgway SH Felten DL Quaranta V. Molecular cloning and characterization of CD4 in an aquatic mammal, the white whale *Delphinapterus leucas*. Immunogenetics. (1999) 49:376–83. 10.1007/s002510050510 10199913

[28] Shirai K Watanabe H Weerasinghe A Sakai T Sekikawa H Abo T. A monoclonal antibody, DL10, which recognizes a sugar moiety of MHC class I antigens expressed on NK cells, NK+ T cells, and granulocytes in humans. J Clin Immunol. (1997) 17:510–23. 10.1023/A:1027379929042 9418192

[29] Shirai K Sakai T Fukuda M Oike T. A monoclonal antibody against dolphin lymphocytes (6E9) which recognizes bovine MHC class II antigens. J Vet Med Sci. (1998) 60:291–3. 10.1292/jvms.60.291 9560774

[30] De Guise S Bernier J Martineau D Beland P Fournier M. Phenotyping of beluga whale blood lymphocytes using monoclonal antibodies. Dev Comp Immunol. (1997) 21:425–33. 10.1016/S0145-305X(97)00021-9 9397348

[31] Romano TA Ridgway SH Quaranta V. MHC class II molecules and immunoglobulins on peripheral blood lymphocytes of the bottlenosed dolphin, *Tursiops truncatus*. J Exp Zool. (1992) 263:96–104. 10.1002/jez.1402630110 1645122

[32] Jaber JR Fernandez A Herraez P Espinosa de los Monteros A Ramirez GA Garcia PM. Cross-reactivity of human and bovine antibodies in striped dolphin paraffin wax-embedded tissues. Vet Immunol Immunopathol. (2003) 96:65–72. 10.1016/S0165-2427(03)00158-2 14522135

[33] Jaber JR Perez J Arbelo M Herraez P Espinosa de los Monteros A Rodnguez F. Immunophenotypic characterization of hepatic inflammatory cell infiltrates in common dolphins (*Delphinus delphis*). J Comp Pathol. (2003) 129:226–30. 10.1016/S0021-9975(03)00008-2 12921729

[34] Beineke A Siebert U Wunschmann A Stott JL Prengel I Kremmer E. Immunohistochemical investigation of the cross-reactivity of selected cell markers from various species for characterization of lymphatic tissues in the harbour porpoise (*Phocoena phocoena*). J Comp Pathol. (2001) 125:311–7. 10.1053/jcpa.2001.0509 11798248

[35] Zabka TS Romano TA. Distribution of MHC II (+) cells in skin of the Atlantic bottlenose dolphin (*Tursiops truncatus*): an initial investigation of dolphin dendritic cells. Anat Rec A Discov Mol Cell Evol Biol. (2003) 273:636–47. 10.1002/ar.a.10077 12808648

[36] Kawashima M Nakanishi M Kuwamura M Takeya M Yamate J. Distributive and phagocytic characteristics of hepatic macrophages in five cetaceans belonging to Delphinidae and Ziphiidae. J Vet Med Sci. (2004) 66:671–80. 10.1292/jvms.66.671 15240942

[37] Schwartz J Aldridge B Blanchard M Mohr FC Stott J. The development of methods for immunophenotypic and lymphocyte function analyzes for assessment of Southern sea otter (*Enhydra lutris nereis*) health. Vet Immunol Immunopathol. (2005) 104:1–14. 10.1016/j.vetimm.2004.06.005 15661326

[38] Elnaggar MM Abdellrazeq GS Venn-Watson SK Jensen ED Hulubei V Fry LM. Identification of monoclonal antibodies cross-reactive with bottlenose dolphin orthologues of the major histocompatibility complex and leukocyte differentiation molecules. Vet Immunol Immunopathol. (2017) 192:54–9. 10.1016/j.vetimm.2017.09.007 29042015

[39] Melnyk PC Romano T editors. Molecular cloning and sequencing of the gene encoding for the cell-surface glycoprotein CD4 from the bottlenose dolphin (*Tursiops truncatus*). In: International Association for Aquatic Animal Medicine. Tampa, FL (2001).

[40] Nouri-Shirazi M Bible BF Zeng M Tamjidi S Bossart GD. Phenotyping and comparing the immune cell populations of free-ranging Atlantic bottlenose dolphins (*Tursiops truncatus*) and dolphins under human care. BMC Vet Res. (2017) 13:78. 10.1186/s12917-017-0998-3 28347312 PMC5369205

[41] Erickson KL DiMolfetto-Landon L Wells RS Reidarson T Stott JL Ferrick DA. Development of an interleukin-2 receptor expression assay and its use in evaluation of cellular immune responses in bottlenose dolphin (*Tursiops truncatus*). J Wildl Dis. (1995) 31:142–9. 10.7589/0090-3558-31.2.142 8583630

[42] Gerner W Stadler M Hammer SE Klein D Saalmuller A. Sensitive detection of Foxp3 expression in bovine lymphocytes by flow cytometry. Vet Immunol Immunopathol. (2010) 138:154–8. 10.1016/j.vetimm.2010.07.009 20701981

[43] Rocchi MS Wattegedera SR Frew D Entrican G Huntley JF McNeilly TN. Identification of CD4+CD25 high Foxp3+ T cells in ovine peripheral blood. Vet Immunol Immunopathol. (2011) 144:172–7. 10.1016/j.vetimm.2011.07.010 21831456

[44] Kaser T Gerner W Hammer SE Patzl M Saalmuller A. Detection of Foxp3 protein expression in porcine T lymphocytes. Vet Immunol Immunopathol. (2008) 125:92–101. 10.1016/j.vetimm.2008.05.007 18565594

[45] Mizuno T Suzuki R Umeki S Okuda M. Crossreactivity of antibodies to canine CD25 and Foxp3 and identification of canine CD4+CD25 +Foxp3+ cells in canine peripheral blood. J Vet Med Sci. (2009) 71:1561–8. 10.1292/jvms.001561 20046022

[46] Beineke A Siebert U van Elk N Baumgartner W. Development of a lymphocyte-transformation-assay for peripheral blood lymphocytes of the harbor porpoise and detection of cytokines using the reverse-transcription polymerase chain reaction. Vet Immunol Immunopathol. (2004) 98:59–68. 10.1016/j.vetimm.2003.10.002 15127842

[47] Hofstetter AR Eberle KC Venn-Watson SK Jensen ED Porter TJ Waters TE. Monitoring bottlenose dolphin leukocyte cytokine mRNA responsiveness by qPCR. PLoS ONE. (2017) 12:e0189437. 10.1371/journal.pone.0189437 29272269 PMC5741220

[48] Sitt T Bowen L Lee CS Blanchard MT McBain J Dold C. Longitudinal evaluation of leukocyte transcripts in killer whales (*Orcinus orca*). Vet Immunol Immunopathol. (2016) 175:7–15. 10.1016/j.vetimm.2016.04.011 27269787

[49] Chen IH Chou LS Chou SJ Wang JH Stott J Blanchard M. Sound exposure-induced cytokine gene transcript profile changes in captive bottlenose dolphin (*Tursiops truncatus*) blood identified by a probe-based qRT-PCR. J Vet Med Sci. (2018) 80:601–5. 10.1292/jvms.17-0548 29479043 PMC5938186

[50] Li WT Wang LY Chang HW Yang WC Lo C Pang VF. Th2 cytokine bias induced by silver nanoparticles in peripheral blood mononuclear cells of common bottlenose dolphins (*Tursiops truncatus*). PeerJ. (2018) 6:e5432. 10.7717/peerj.5432 30245924 PMC6147119

[51] Fonfara S Siebert U Prange A Cytokine and acute phase proteins as markers for infection in harbour porpoises (*Phocoena phocoena*). Mar Mammal Sci. (2007) 23:931–42. 10.1111/j.1748-7692.2007.00140.x

[52] Beineke A Siebert U Muller G Baumgartner W. Increased blood interleukin-10 mRNA levels in diseased free-ranging harbor porpoises (*Phocoena phocoena*). Vet Immunol Immunopathol. (2007) 115:100–6. 10.1016/j.vetimm.2006.09.006 17055589

[53] Eberle KC Waters TE Jensen ED Venn-Watson SK Sacco RE. Development and application of specific cytokine assays in tissue samples from a bottlenose dolphin with hyperinsulinemia. Front Endocrinol. (2013) 4:134. 10.3389/fendo.2013.00134 24101915 PMC3787309

[54] Soloff AC Wolf BJ White ND Muir D Courtney S Hardiman G. Environmental perfluorooctane sulfonate exposure drives T cell activation in bottlenose dolphins. J Appl Toxicol. (2017) 37:1108–16. 10.1002/jat.3465 28425113 PMC5831401

[55] Jaber JR Perez J Zafra R Herraez P Rodriguez F Arbelo M. Cross-reactivity of anti-human, anti-porcine and anti-bovine cytokine antibodies with cetacean tissues. J Comp Pathol. (2010) 143:45–51. 10.1016/j.jcpa.2010.01.001 20163803

[56] Diaz-Delgado J Ressio R Groch KR Catao-Dias JL. Immunohistochemical investigation of the cross-reactivity of selected cell markers in formalin-fixed, paraffin-embedded lymphoid tissues of Franciscana (*Pontoporia blainvillei*). Vet Immunol Immunopathol. (2018) 200:52–8. 10.1016/j.vetimm.2018.04.009 29776612

[57] Funke C King DP McBain JF Adelung D Stott JL. Expression and functional characterization of killer whale (*Orcinus orca*) interleukin-6 (IL-6) and development of a competitive immunoassay. Vet Immunol Immunopathol. (2003) 93:69–79. 10.1016/S0165-2427(03)00055-2 12753777

[58] Eberle KC Venn-Watson SK Jensen ED LaBresh J Sullivan Y Kakach L. Development and testing of species-specific ELISA assays to measure IFN-gamma and TNF-alpha in bottlenose dolphins (*Tursiops truncatus*). PLoS ONE. (2018) 13:e0190786. 10.1371/journal.pone.0190786 29304133 PMC5755893

[59] Levin M Romano T Matassa K De Guise S. Validation of a commercial canine assay kit to measure pinniped cytokines. Vet Immunol Immunopathol. (2014) 160:90–6. 10.1016/j.vetimm.2014.04.001 24845148

[60] Canavan JB Afzali B Scotta C Fazekasova H Edozie FC Macdonald TT. A rapid diagnostic test for human regulatory T-cell function to enable regulatory T-cell therapy. Blood. (2012) 119:e57–66. 10.1182/blood-2011-09-380048 22219224 PMC3816350

[61] Gerriets VA Kishton RJ Johnson MO Cohen S Siska PJ Nichols AG. Foxp3 and Toll-like receptor signaling balance Treg cell anabolic metabolism for suppression. Nat Immunol. (2016) 17:1459–66. 10.1038/ni.3577 27695003 PMC5215903

[62] Michalek RD Gerriets VA Jacobs SR Macintyre AN MacIver NJ Mason EF. Cutting edge: distinct glycolytic and lipid oxidative metabolic programs are essential for effector and regulatory CD4+ T cell subsets. J Immunol. (2011) 186:3299–303. 10.4049/jimmunol.1003613 21317389 PMC3198034

[63] Chen Y Colello J Jarjour W Zheng SG. Cellular metabolic regulation in the differentiation and function of regulatory T cells. Cells. (2019) 8:188. 10.3390/cells8020188 30795546 PMC6407031

[64] Hori S Nomura T Sakaguchi S. Control of regulatory T cell development by the transcription factor Foxp3. Science. (2003) 299:1057–61. 10.1126/science.1079490 12522256

[65] Cote-Sierra J Foucras G Guo L Chiodetti L Young HA Hu-Li J. Interleukin 2 plays a central role in Th2 differentiation. Proc Natl Acad Sci USA. (2004) 101:3880–5. 10.1073/pnas.0400339101 15004274 PMC374338

